# Surgical Therapeutic Strategy for Non-small Cell Lung Cancer with Mediastinal Lymph Node Metastasis (N2)

**DOI:** 10.3779/j.issn.1009-3419.2010.04.14

**Published:** 2010-04-20

**Authors:** Qianli MA, Deruo LIU, Yongqing GUO, Bin SHI, Zhiyi SONG, Yanchu TIAN

**Affiliations:** Department of Thoracic Surgery, China-Japan Friendship Hospital, Beijing 100029, China

**Keywords:** Lung neoplasms, Thoracic surgical procedures, Lymphatic metastasis, Treatment outcome

## Abstract

**Background and objective:**

Approximately 30% of patients who are diagnosed with non-small cell lung cancer (NSCLC) are classified as N2 on the basis of metastasis to the mediastinal lymph nodes. The effectiveness of surgery for these patients remains controversial. Although surgeries in recent years are proved to be effective to some extent, yet due to many reasons, 5-year survival rate after surgery varies greatly from patient to patient. Thus it is necessary to select patients who have a high probability of being be cured through an operation, who are suitable to receive surgery and the best surgical methods so as to figure out the conditions under which surgical treatment can be chosen and the factors that may influence prognosis.

**Methods:**

165 out of 173 patients with N2 NSCLC were treated with surgery in our department from January 1999 to May 2003, among whom 130 were male, 43 female and the sex ratio was 3:1, average age 53, ranging from 29 to 79. The database covers the patients' complete medical history including the information of their age, sex, location and size of tumor, date of operation, surgical methods, histologic diagnosis, clinical stage, post-operative TNM stage, neoadjuvant treatment and chemoradiotherapy. The methods of clinical stage verification include chest X-ray, chest CT, PET, mediastinoscopy, bronchoscope (+?), brain CT or MRI, abdominal B ultrasound (or CT), and bone ECT. The pathological classification was based on the international standard for lung cancer (UICC 1997). Survival time was analyzed from the operation date to May 2008 with the aid of SPSS (Statistical Package for the Social Sciences) program. *Kaplan-Meie*r survival analysis, *Log-rank* test and *Cox* multiplicity were adopted respectively to obtain patients' survival curve, survival rate and the impact possible factors may have on their survival rate.

**Results:**

The median survival time was 22 months, with 3-year survival rate reaching 28.1% and 5-year survival rate reaching 19.0%. Age, sex, different histological classification and postoperative chemoradiotherapy seem to have no correlation with 5-year survival rate. In all N2 subtypes, 5-year survival rate is remarkably higher for unexpected N2 discovered at thoractomy and proven N2 stage before preoperative work-up and receive a mediastinal down-staging after induction therapy (*P* < 0.01), reaching 30.4% and 27.3% respectively. 5-year survival rate for single station lymph node metastasis were 27.8%, much higher compared with 9.3% for multiple stations (*P* < 0.001). Induction therapy which downstages proven N2 in 73.3% patients gains them the opportunity of surgery. The 5-year survival rate were 23.6% and 13.0% for patients who had complete resection and those who had incomplete resection (*P* < 0.001). Patients who underwent lobectomy (23.2%) have higher survival rate, less incidence rate of complication and mortality rate, compared with pneumonectomy (14.8%) (*P* < 0.01). T4 patients has a 5-year survival rate as low as 11.1%, much less than T1 (31.5%) and T2 (24.3%) patients (*P*=0.01). It is noted through *Cox* analysis that completeness of resection, number of positive lymph node stations and primary T status have significant correlativity with 5-year survival rate.

**Conclusion:**

It is suggested that surgery (lobectomy preferentially) is the best solution for T1 and T2 with primary tumor have not invaded pleura or the distance to carina of trachea no less than 2 cm, unexpected N2 discovered at thoractomy when a complete resection can be applied, and proven N2 discovered during preoperative work-up and is down-staged after induction therapy. Surgical treatment is the best option, lobectomy should be prioritized in operational methods since ite rate of complication and morality are lower than that of pneumonectomy. Patients' survival time will not benefit from surgery if they are with lymph nodes metastasis of multiple stations (Bulky N2 included) and T4 which can be partially removed. Neoadjuvant chemotherapy increases long-term survival rate of those with N2 proven prior to surgery. However, postoperative radiotherapy decreases local recurrence rate but does not contribute to patients' long-term survival rate.

## Introduction

Approximately 30% of patients who are newly diagnosed with non-small cell lung cancer (NSCLC) are classified as N2 on the basis of metastasis to the mediastinal lymph nodes. Their median survival time is 7 months. Chemoradiation therapy has limited effect. Surgery may bring about some positive results, yet the 5-year survival rate of different patients varies greatly. Factors leading to the differences are ways to verify N2 (pre-surgery radiology, pathology before, during and after surgery), the degradation state of mediastinal lymph node metastasis after chemotherapy, grade of resection, surgical methods, types of pathology, location and size of tumor, numbers of lymph nodes and post-operative adjuvant therapy. Therefore, it is necessary to select patients who have a high probability of being be cured through an operation, who are suitable to receive surgery and the best surgical methods. In this paper, N2 is classified into 4 subgroups. The first group is unexpected N2 (incidental or surprised N2), which belongs to period Ⅰ or Ⅱ clinical stage classification, yet is found out to be N2 matastasis through pathology after operation or single station mediastinal lymph node metastasis during operation. The second group is marginal or proven N2. There is mediastinal mass-like density on the basis of radiology before surgery, and single or multiple lymph node matastasis proven through mediastinotopy, PET/ CT, or lymph biopsy. The third group is Bulky N2 with short radius more than 2 cm or lymph matastasis of multiple stations. The fourth group is N2 with primary tumors as T4. We retrospectively analyzed the effect of the operations we had on 173 patients at N2 period from 1999 to 2003, so as to identify the surgical indication and evaluate the prognostic factors.

## Common Data

380 patients with primary tumors had surgeries at thoracic surgery of China-Japan Friendship Hospital from January 1999 to May 2003. Among them, 173 were proven N2 before or after the surgery through pathology, 165 of them had operation and 13 exploratory thoracotomy (7.9%). With 130 male and 43 female, the sex ratio was 3:1. The average age of the patients were 53, ranging from 29 to 79. There were patients' follow-up data and complete medical history including the information of their age, sex, location and size of tumor, date of operation, surgical methods, histological diagnosis, clinical stage, pathologic type, post-operative TNM stage, pre-surgical chemotherapy, degradation after chemotherapy, cycles of chemotherapy after operations, radio therapy after operation, viable status and survival time. The lost to follow-up rate was 13%. The methods of clinical stage verification include Posterior to Anterior (PA) chest X-ray, chest CT, PET, mediastinoscopy, bronchoscope, brain CT, abdominal B ultrasound, and bone ECT. The patho-logical classification was based on the international standard for lung cancer (UICC 1997).

## Statistical analysis

Survival time was analyzed from the operation date to May 2008 with the aid of SPSS (Statistical Package for the Social Sciences) program. *Kaplan-Meier* survival analysis, *Log-rank* test and *Cox* multiplicity were adopted respectively to obtain patients' survival curve, survival rate and the impact possible factors may have on their survival rate.

## Results

### Post-operative survival status

The median survival time was 22 months, with the 3-year and 5-year survival rates reaching 28.1% and 19.0% respectively.

### The correlation between patient characteristics and prognosis

Factors such as age, gender, pathologic type, post-operative chemotherapy, post-operative radiotherapy appear to have a few relation with patients' 5-year survival time (Tab 1). 5-year survival rate for those who had lobectomy was 23.6%, substantially exceeding 13.0%, the rate of those who had pneumonectomy (*P* < 0.001) among (25%), the survival curve is showed in Fig 1. 5-year survival rate for T4 patients were 11.1%, much lower than that of T1 (31.5%) and T2 (24.3%) (*P*=0.01). This survival curve is in Fig 2. It can be seen in Tab 2 that the ratio of single station lymph node and the rate of complete resection of subgroup Ⅰ and Ⅱ are higher than that of subgroup Ⅲ and Ⅳ. 5-year survival rate for the unexpected were 30.4%, and that of N2 stage proven before preoperative work-up and receive a mediastinal down-staging after induction therapy were 27.3%, significantly higher than other N2 subtypes (*P* < 0.01). Induction therapy which downstages proven N2 in 73.3% patients gain them the opportunity of surgery. It is illustrated in Tab 3 that the 5-year survival rate of patients who had lobectomy was (23.2%) have higher survival rate, less incidence rate of complication and mortality rate, compared with pneumonectomy (14.8%).

**1:** Relationship between the clinical data of 173 NSCLC cases and the long-time survival rate

Factors	Cases (%)	Survival rate (%)		*P*
		3-year	5-year	
Age				0.88
≤60	84 (48.6)	22.2	27.8	
< 60	89(51.4)	33.3	12.5	
Sex				0.15
Male	130(75.1)	25.5	12.1	
Female	43 (24.9)	40.0	44.4	
Operation				< 0.001
Complete resection	110(66.7)	39.4	23.6	
Uncertain resection	19(11.5)	44.4	21.1	
Uncomplete resection	23 (13.9)	25.3	13.0	
Explore	13 (7.9)	15.4	7.7	
T-stage				0.01
T1	10 (5.8)	47.9	31.5	
T2	58 (33.5)	38.4	24.3	
T3	59 (34.1)	32.2	18.4	
T4	46 (26.6)	28.6	11.1	
Lymphnodes metastasis				< 0.001
Single station	91 (52.6)	48.4	27.8	
Multiple stations	82 (47.4)	19.5	9.3	
Pathological classification				0.753
Adenocarcinoma	77 (44.5)	11.1	8.3	
Squamous carcinoma	64 (37.0)	42.9	25	
Mixed carcinoma	16 (9.2)	25	0	

**1:**
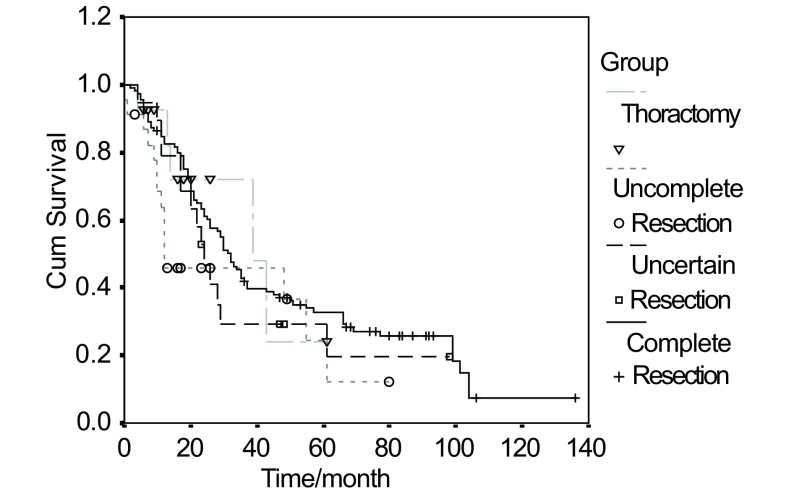
*Kaplan-Meier* analysis of survival rates based on operation methods for 173 patients with N2 NSCLC diseases

**2:**
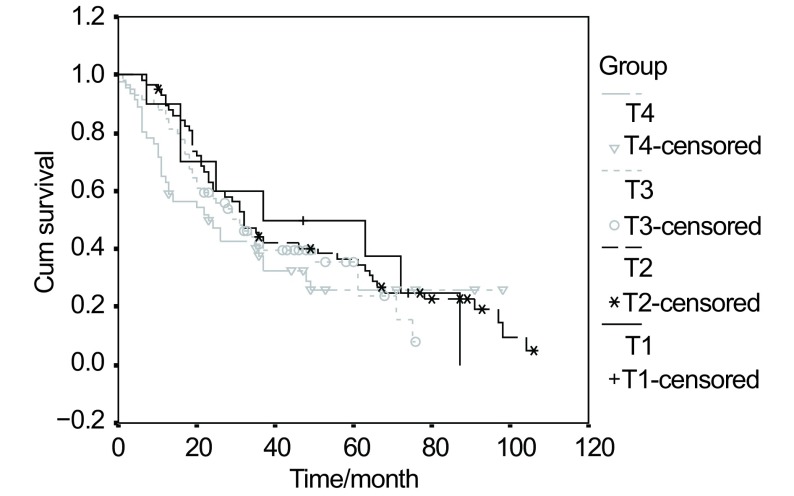
*Kaplan-Meier* analysis of survival rates based on T status for 173 patients with N2 NSCLC diseases

**2:** Comparison of different subtypes in 173 NSCLC-N2 cases

	Unexpected	IIIa(T1, T2, T3) Proven			Bulky	IIIb (T4)
		NAT+O	O	NAT		
Cases	46	22	39	8	12	46
1 station	34 (73.9%)	36 (52.2%)	2 (16.7%)	19 (41.3%)		
> 2 station	12 (26.1%)	33 (47.8%)	10 (83.3%)	27 (58.7%)		
Complete resection	40 (87.0%)	47 (77.0%)	3 (25.0%)	20 (43.8%)		
5-year survival (%)	30.4	27.3	17.9	12.5	0.0	11.1%

**3:** Comparison of lobectomy and pneumonectomy

	Lobotectomy	Pneumonectomy
Cases	125	27
5-year survival rate (%)	23.2	14.8
Percentage of T4 (%)	16.0	44.4
△FEV1 ↓	14.7%	32.3%
Pneumonia	9 (7.2%)	3(11.1%)
Respiratory failure	7 (5.6%)	2 (7.4%)
Heart failure	5 (4.0%)	3(11.1%)
Bleed	5 (4.0%)	2 (7.4%)
Pulmonary embolism	4 (3.2%)	2 (7.4%)
Arrhythmia	20(16.0%)	3 (11.1%)

### Factors influencing prognosis

It is noted through *Cox* analysis that completeness of resection, classification of T status and number of positive lymph node stations and have significant correlativity with 5-year survival rate (Tab 4).

**4:** Multivariate *Cox* proportional Hazard Analysis of 5-year survival rate

Variable	Regression coefficient	Relative risk	95%CI	*P*
Complete resection	-0.903	0.407	0.206-0.797	0.009
Uncomplete resection	0.752	2.03	1.051-3.814	0.039
T4	0.646	1.909	1.020-3.572	0.043
T3	0.053	1.054	0.545-2.038	0.875
Signal station	-0.932	0.374	0.185-0.772	0.008
Multiple stations	0.551	1.786	0.874-3.129	0.052

## Discussion

The prognosis of N2 stage NSCLC is unfavorable, and the outcome of surgery is limited, so the treatment strategy is highly controversial all over the world. We evaluated several factors in our series and compared them with other reports in order to decide surgical indication and provide the best combined therapy. N2 NSCLC belongs to locally advanced cancer and has a 7-month natural median survival time. Patients who are not suitable to resection, the standard treatment pattern is chemotherapy (platinum contained) plus radiotherapy. Median survival time with synchronic chemoradiation therapy is 17 months, with 5-year survival rate reaching 16%. Median survival time with sequential chemoradiation therapy is 13 months, and its 5-year survival rate is 9%^[1]^. In order to maximize the long-term rate of N2 patients, we need to explore proper surgical treatment. Combined with related report, our series is to analyze the result of our survey so as to probe into the factors influencing prognosis and possible reasons, as well as clarifying conditions under which surgical options are made.

### Clinical characteristics and prognosis

The 5-year survival rate in those whose age was above 60 years (12.5%) was less than that of patients whose age were under 60 years (27.8%), *P*=0.88. The survival rate for male (12.1%) was less than female (44.4%), but the percentages have no statistic value, *P*=0.15. Just as Vansteenkiste *et al*^[2]^ reported, age and gender cannot be seen as independent factors decisive to the prognosis.

### Surgical treatment and Prognosis

Theoretically, N2 NSCLC patients could be classified as resectable and unresectable by pre-surgical evaluation. The resectable group covers the following subgroups. First, the unexpected N2 (Cases classified as stage Ⅰ, Ⅱ of CTNM before operation then discovered with mediastinal lymph node metastasis after operation). Second, single or multiple mediastinal lymph node metastasis testified through radiology and could be fully removed (The key point of distinguishing whether the enlarge lymphondes invade normal structures or not is to see if there is a low density zone between them through chest CT. The zone always indicates a layer of fat and all lymphondes can always be completely eliminated after soThly inactively dissection during operation). Third, a portion of T4 cases. The unresectable group includes N2 proven before surgery, the positive cases with mediastinal mass-like density shown by radiology, and proven by mediastinoscopy (edge Ⅲ stage) and most of T4.

In term of the surgical character, 4 types can be classified: (1) Complete resection: 4 conditions must be meted. First, all the incisal edges (including the bronchus, artery, vein, tissues around the bronchus and the primary tumor) are negative (free of tumor cells) under the microscope. Second, systematic or lobe systematic lymph nodes dissection should be done, and 6 groups must contained, 3 locate in the lobe (lobar, interlobe or segmental lymph node) and hilar, the other 3 are mediastinal lymph nodes (subcarinal included). Third, no invasion is found out of the lymph nodes in the dissected mediastinal or lobe marginal nodes. Fourth, the highest mediastinal lymph nodes must be resected, and should be negative under the mictoscope. (2) Uncomplete resection: tumor cells were left on the cutting edge, invasion out of the lymph nodes was found in mediastinal or lobe's marginal lymph nodes. Lymph nodes were positive with tumor cells, but cannot be dissected, carcinoma cells were found in the pleura or pericardium effusion. (3) Uncertain resection: no evidence of the residual tumor, but the operation can not meet the criterion of complete resection, that is, all the cut-edge are negative under the microscope, but one of the following situations come up, carcinoma in situ is found on the cut-edge, lymph nodes dissection can not meet the criterion mentioned above, the highest mediastinal lymph nodes are positive but are already be dissected, tumor cells are found in the washing liquor from the pleura cavity. (4) Explore: the thoracic was only opened, but tumor tissues can not be resected or only biopsy was applied.

The retrospective research of our series indicates that for N2 cases selected above, the rate of resection was 66.7%, 5-year survival rate was 23.6%, significantly higher than that of surgery of other natures, and the survival curve was show in Fig 1.

Surgeries consist of lobectomy and pneumonectomy, The analysis of this series suggests that patients who had lobectomy (104 standard operations, 12 sleeve resection included and 9 lobectomy under thoracoscope) have a higher 5-year survival rate of 23.2% than 14.8% for patients who had pneumonectomy (*P* < 0.01).

We attribute this to following factors. First, pneumonectomy compromise more lung factions (Tab 3. Six months after the lobectomy, FEV1 was reduced by 14.7%, pneumonectomy dropped by 32.3%). Second, T stage of primary tumor in pneumonectomy was later; the rate of T4 rate was enormously increased (44%). Third, the rate of complication for pneumonectomy was higher, including pneumonia (11.1%), respiratory failure (7.4%), heart failure (11.1%), bleeding (7.4%), pulmonary embolism (7.4%), cardiac arrhythmia (11.1%). Kiser^[3]^ also verified that resection is a risk factor for prognosis. Therefore, with techniques workable/available, sleeve resection instead of pneumonectomy is recommended.

### T status and prognosis

Mountain^[4]^and Regnard *et al*^[5]^reported that the earlier the T stage, the better the prognosis. In our series research, the surgical indication for N2 NSCLC was based on two criteria: no invasion of the pleura by the tumor, a distance between the margin of the tumor and carina of more than 2 cm (T1 and T2). Patients belonging to this stage had a better 5-year survival rate (37.5%) than T4 patients (11.1%, *P*=0.01).

The criterion of choosing T4 patients for operation are as follows: (1) The pulmonary artery, vein, bronchus and these branches are evaluated can be dissected through chest enhancement CT. (2) Sleeve resection can be carried out through the observation from bronchoscope (mostly upper lobe sleeve resection for squamous carcinoma). (3) No more than 1/3 of the SVC (superior vena cava) is invaded, and then the blood vessel plasty by patch or reconstruction operation can be done. (4) The atrium is invaded, but the defected part can be sewed up or patched by a part of pericardium. (5) Only the external membrance of the aorta is invaded, the function of blood vessel's wall is basically not affected after tumor removed. (6) Metastasis nodes are found in the same lobe. The specific distribution of the cases: carina invaded (*n*=10), SVC (*n*=4), atrium (*n*=7), aorta (*n*=3), metastasis nodes are found in the same lobe (*n*=8). Invasion of the vertebra, esophagus, trachea and maligment pleura effusion are nit included.

If patients could be selected with the consideration of the standard above, surgery can reduce tumor, mitigate coughing, chest pain and chest tightness, improving patients' living quality. However, since the rate of uncomplete resection (15.6%) is much higher than that of T1/T2 (2%) and T3 (5.9%), it has very limited effect to long-term survival rate. Therefore, surgery is not a proper option for T4 patents and needs to be adopted carefully.

### Medastinal lymph node metastasis and prognosis

Ishida^[6]^ figured out through research that probabilities of lymph node metastasis for tumors with radius lesser than 1 cm, 1.1 cm to 2 cm, 2.1 cm to 3 cm are 0%, 17% and 38% respectively. The standard of pheumoecotomy requires not only lobecotomy where primary tumor is, but also the complete removal of mediastinal lymph node, otherwise the recurrence rate will be increased.

In this paper, N2 is classified into 4 subgroups. The first group is Unexpected N2 (incidental or surprised N2), which belongs to period Ⅰ or Ⅱ clinical stage classification, yet is found out to be N2 metastasis through pathology after operation or single station mediastinal lymph node metastasis during operation. The second group is marginal or proven N2. There is mediastinal mass-like density on the basis of radiology before surgery, and single or multiple lymph node metastasis proven through mediastinoscopy, PET/CT, or lymph biopsy. The third group is Bulky N2 with short radius more than 2 cm or stable lymph matastasis of multiple stations. The fourth group is N2 with primary as T4. The percentages of single station lymph node and the rate of pneumoecotomy is much higher for unexpected N2 and proven N2 discovered before surgery and is down-staged by neoajuvant therapy, compared with bulky N2 and N2 with primary tumor as T4. 5-year survival rates for unexpected N2 and N2 proven and down-staged ahead of surgery are 30.4 % and 27.3%, substantially higher than other N2 types (*P* < 0.01). 5-year survival rate for singlestation lymph node metastasis is 27.8%, significantly higher than 9.3% for multiple-station one (*P* < 0.01).

Patients with single mediastinal N2 featuring in enlarged and removable lymph node are recommended to apply a combination of neoadjuvant chemotherapy, surgery chemotherapy-or radiotherapy, which is consistent with Kara's^[7]^ view. For multiple-station N2 or bulky N2 could be divided into resectable and unresectable ones. The effect after the resection is unfavorable and non-surgical methods should dominate the treatment.

### Pathological characteristics and prognosis

Arguments still exists on the relation of these two factors. It is reported that adenocarcinoma cells are prone to metastasis (v) through blood and lymph systems, the long-term survival time of squamous cell carcinoma patients was longer than other patients (adenocarcinoma included). However, in the perspective of statistics, there was no difference in survival time for those with squamous cell carcinoma (25.0%) and those adenocarcinoma (8.3%) in our study (*P* > 0.05). Additionally, the rate for mixed carcinoma, big-cell cancer and others were 0.0%, 0.0% and 33.3%. The reason is still unknown, but one likely explanation may be that follow-up was conducted on a limited number of patients.

### Adjuvant therapy and prognosis

#### Pre-surgical chemotherapy

The current randomized controlled studies shows that presurgical neoadjuvant is beneficial to long-term survival for N2 patients. There are 5 RCTs which illustrate that the 5-year survival rate for those who had chemotherapy before their surgeries were 28%, yet that of those who had surgeries only were 16%. The difference in 2 related studies has statistical indication^[8]^.

The reason for this is that chemotherapy can downgrade the stage of tumors, thus facilitates the radical treatment. Pre-surgical chemotherapy could raise the 5-year survival rate from 14% to 20%^[9]^, according to the *meta* analysis in 12 clinical research conducted by Burddet in 2006. It is proved in our research that pre-surgical neoadjuvant chemotherapy can down-stage N2 status and gain patients chances of operations, increasing their 5-year survival rate from 17.9% to 27.3%.

#### Post-surgical chemotherapy

It is arguable whether patients should have chemotherapy after surgeries and how many chemotherapy are reasonable. Most people agree that consolidate the chemotherapy scheme is necessary. Nevertheless, Scoinski^[10]^ regards that extending post-surgical periods can not improve survival time. On contrary, it increases the toxics of chemotherapy and compromise quality of patients' life.

Socinski^[10]^ reports that the 5-year survival rate of locally advanced NSCLC with the 3^rd^ generation of cisplatin-based chemotherapy after surgeries is 37%, higher than 32%, the rate of those without the chemotherapy (*P* < 0.05). Similarly, researches done by ANITA^[11]^ indicates that NP scheme adjvant chemotherapy prolongs patients' survival time.

In our series, 101 patients underwent post-surgical chemo-therapy, 5-year survival rate was 35% for those had 4 durations of post-chemotherapy, higher than 18.8%, the rate of those without the post-surgical therapy. The patients who had postsurgical chemotherapy lasting less or more than 4 durations were 14.3% and 333.3% respectively. However, these percentages don't have statistical indication. It suggests that post-surgical chemotherapy did not play an effective role in reducing the recurrence rate and raising survival rate without recurrence recurrence-free survival rate. According to the result from 3 random clinical comparative research conveyed by Socinski^[12]^, for those who had 2-4 durations of chemotherapy and those who had 6 or more durations of chemotherapy, the survival rates are almost the same. Moreover, the toxic and side effects of chemotherapy will be reduced by limit the number of durations and it is recommended that 4 duration chemotherapy after operation should be the optical solution.

It is worthy of notice that pneumoectomy, especially the right pneumoectomy takes a long time for recovery and is not suitable for adjuvant therapy. This view is shared by Yilon Wu^[13]^.

#### Radiotherapy

In our series, 5-year survival rate of the patients with and without the radiotherapy were 6.3% and 26.9% (*P*=0.317). Despite of the disparity in the percentages, there is no obvious difference in statistic views in that patients who had palliative resection accounted for a large percentage of the overall patients in the post-surgical radiotherapy group. Local recurrence rate as high as 19% to 29% was seen in patients who had non-standard dissection of lymph nodes (the number of dissections is less than 3 stations, especially with the 7^th^ lymph node included), extra-capsular spread/invasion in lymph node, multiple N2 metastasis and less than 2 cm's distance between tumor and resection margin of bronchus. They are high risk group of patients^[14]^.

Cochrane system, conversely, it is proved that post-surgical radiotherapy reduces the local recurrence rate, yet it has negative influence on survival rate and increases the risk of death by 21%, which is equal to cutting down the 2-year survival rate by 7%, lowering the overall survival rate from 55% to 48%^[15]^.

Whereas Sawyer^[16]^ proposes the idea that post-surgical radiotherapy may improve long-term survival time for N2 patients, hence whether post-surgical radiotherapy can increase overall survival rate remains controversial for those who had incomplete resection. Now that adjuvant radiotherapy brings down overall survival rate, it is not recommended to the patients with complete resection.

In summary, we have reached the conclusion that among the factors influencing prognosis of N2 NSCLC patients, surgery is the best option for T1 and T2 patients whose primary tumor had been detected before surgery with the tumor not invading the pleura and the distance between the tumors and tracheal carina is no less than 2 cm, patients who are with negative mediastinal lymph node and found single station N2 removable during surgery, patients with N2 proven before surgery but down-staged by neoaduevant chemotherapy. The best surgical type is lobectomy which cause less incidence of complication and death compared with pneumoectomy. Patients' survival time will not benefit from surgery if they are with lymph nodes metastasis of multiple stations (Bulky N2 included) and T4 which can be partially removed. Neoadjuvant chemotherapy increases long-term survival rate of those with N2 proven prior to surgery. However, post-surgical radiotherapy decreases local recurrence rate but does not contribute to patients' long-term survival rate.

## Acknowledgement

This study was supported by grants from the Science and Research Department, belongs to China-Japan Friendship Hospital directly affiliated to Chinese Ministry of Health.
